# Comparison of Real-Time PCR, Bacteriologic Culture and Fluorescent Antibody Test for the Detection of *Leptospira borgpetersenii* in Urine of Naturally Infected Cattle

**DOI:** 10.3390/vetsci7020066

**Published:** 2020-05-15

**Authors:** Jarlath E. Nally, Ahmed A. A. Ahmed, Ellie J. Putz, Debra E. Palmquist, Marga G. A. Goris

**Affiliations:** 1 Infectious Bacterial Diseases, National Animal Disease Center-ARS-USDA, 1920 Dayton Avenue, Ames, IA 50010, USA; Ellie.Putz@usda.gov; 2 OIE and National Collaborating Centre for Reference and Research on Leptospirosis, Academic Medical Center, University of Amsterdam, 1105 AZ Amsterdam, The Netherlands; a.ahmed@amsterdamumc.nl (A.A.A.A.); m.goris@amsterdamumc.nl (M.G.A.G.); 3 Midwest Area Statistics Unit, ARS-USDA, 1815 North University St., Peoria, IL 61604, USA; deb.palmquist@usda.gov

**Keywords:** cattle diseases, *Leptospira*, leptospirosis, urine

## Abstract

Cattle are susceptible to infection with multiple serovars of pathogenic leptospires, resulting in abortion, stillbirth, premature birth, reproductive failure and milk drop syndrome. Cattle also act as a reservoir host for *L. borgpetersenii* serovar Hardjo which is excreted from renal tubules via urine into the environment where it persists in suitable moist conditions. Our previous work demonstrated that 7% of urine samples from beef cattle were positive for *L. borgpetersenii* serovar Hardjo by culture and/or the fluorescent antibody test (FAT). In this study, a real-time PCR (rtPCR) assay was applied to determine the relative performance of rtPCR based detection of *L. borgpetersenii* serovar Hardjo compared to previously reported culture and FAT techniques. Of 42 bovine urine samples positive for leptospires by culture and/or FAT, 60% (25/42) were positive by rtPCR. Of 22 culture-positive samples, 91% (20/22) were rtPCR-positive. Of 32 FAT-positive samples, 50% (16/32) were rtPCR-positive. For 10 samples that were culture-positive but FAT-negative, 90% (9/10) were rtPCR-positive. For 20 samples that were FAT-positive but culture-negative, 25% (5/20) were rtPCR-positive. Collectively, these results indicate that no single assay is optimal, and the use of more than one assay to detect leptospires in urine from naturally infected cattle is recommended.

## 1. Introduction

Leptospirosis is a global zoonotic disease [1]. Pathogenic *Leptospira* colonize the renal tubule of reservoir hosts of infection, including domestic and wild animal species, from where they are excreted via urine into the environment and persist in suitable moist conditions [2]. Contact with contaminated environmental sources, or directly with urine from infected animals, can result in acute infection in incidental hosts, as pathogenic *Leptospira* can penetrate mucosal surfaces or breaches of the skin. Over 1 million cases of human disease are estimated to occur annually, with almost 60,000 deaths [3]. Leptospirosis is also a significant cause of morbidity and mortality in domestic animals, including cattle, dogs, sheep, pigs and horses, which can be both incidental and reservoir hosts, depending on the species and serovar of *Leptospira* involved [4]. Clinical symptoms range from a mild fever to more severe icteric disease and massive pulmonary hemorrhage, reflecting systemic dissemination of different serovars throughout the host. Animal and human patients that suffer acute leptospirosis may continue to shed leptospires in urine despite the clinical resolution of symptoms [5,6,7]. In domestic animals, the greatest economic losses arise from chronic infection, causing reproductive wastage [4]. Disease transmission of all pathogenic *Leptospira* is maintained by asymptomatic reservoir hosts of infection where a unique biological equilibrium exists between specific animal hosts and specific serovars of *Leptospira*, and as exemplified by *Leptospira borgpetersenii* serovar Hardjo in bovine populations throughout the world [8,9].

Bovine leptospirosis can result in abortion, stillbirth, premature birth, reproductive failure and milk drop syndrome [4]. Cattle are susceptible to infection with multiple *Leptospira* species and serovars including *L. borgpetersenii* serovar Hardjo, *L. interrogans* serovar Pomona, *L. kirschneri* serovar Grippotyphosa and *L. noguchii* [10,11,12]. However, the most prominent serovar associated with cattle is Hardjo, which causes reproductive failure [8,11,13]. In cows seropositive for Hardjo, the median time from calving to conception (132.6 days) was significantly longer than time for seronegative cows (95.4 days) [14]. Cows that were seropositive to serovar Hardjo were twice as likely to fail to conceive as seronegative cows. Seroprevalence studies indicate that up to 49% of cattle are exposed to pathogenic serovars [11]. Seronegative animals may also excrete *Leptospira* [11,12].

**Figure 1:**
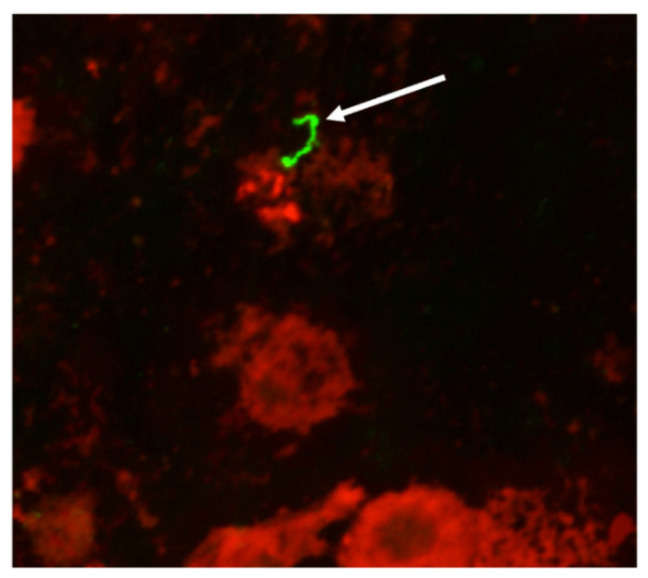
Representative bovine urine sample positive for leptospires by fluorescent antibody test (FAT). Arrow indicates the expected morphology of *Leptospira* detected in a bovine urine sample. Original magnification 400×.

The definitive assay to identify cattle that are shedding leptospires in urine is culture, which results in an isolate of *Leptospira* that can be completely characterized at the genetic and serovar level, and is readily available for use in microscopic agglutination test (MAT) diagnostic panels or inclusion in bacterin-based vaccines. However, culture can take weeks to months, and requires highly specialized media. Alternatively, the fluorescent antibody test (FAT) can be performed relatively quickly using antibodies that provide specificity for the detection of pathogenic leptospires as well as visual confirmation of the morphology of intact leptospires actively excreted in urine (Figure 1) [12]. However, the FAT does not provide serovar or species identification. Molecular assays such as PCR can be performed relatively quickly and are used to infer the presence of leptospires in urine samples; advantages include sensitivity, quantification, and the ability to sequence amplified products that can be used to identify the pathogenic species involved [15,16]. A range of factors can influence the choice of assay used to detect the presence of leptospires in urine samples, including the availability of resources, skillset, time, diagnostic goals and downstream applications. It has previously been reported that the use of at least two detection techniques was required to detect *L. borgpetersenii* serovar Hardjo in the urine of experimentally infected cattle [17].

Our recent work demonstrated that 7% (43/600) of sampled beef cattle were actively shedding leptospires in urine as detected by culture and/or FAT [12]. Of the 43 test-positive samples, 13 were positive by both culture and FAT, whereas 10 were positive only by culture, and 20 were positive only by FAT. In the current study, we assessed the benefit of applying an alternative molecular real-time PCR (rtPCR) assay to detect leptospires in 42 of these test-positive bovine urine samples to determine relative performance of each assay in samples from naturally infected cattle.

## 2. Materials and Methods

Bovine urine sample collection and processing was as previously described for FAT [12], except that the final washed urine pellet was stored at −80 °C until total nucleic acid extraction was performed using an automated system (NucliSENS easyMAG, BioMérieux, St. Louis, MO, USA), according to manufacturer’s instructions. In brief, the urinary pellet was resuspended in 2 mL of easyMAG lysis buffer for at least 20 min before input to the automated extraction system. DNA was eluted in a final volume of 80 μL. A previously published SYBR Green real-time PCR, which was validated according to OIE requirements [18], targeting *secY* with the primer set SecYIVF (5′GCGATTCAGTTTAATCCTGC′3) and SecYIV (5′GAGTTAGAGCTCAAATCTAAG′3), was performed as previously described, including melting curve construction and melting temperature value determination [16]. Of the 43 original bovine urine culture/FAT-positive samples collected [12], only 42 were available and all were processed in quadruplicate, as well as 10 additional negative control urine samples derived from cattle that were MAT-, FAT- and culture-negative for leptospires.

Percent observed agreement was determined between the rtPCR method of detection and the results of previously reported culture and FAT detection methods. For both comparisons (rtPCR and culture, rtPCR and FAT), the total shared positive and negative samples were evaluated. Cohen’s Kappa was evaluated to assess the pairwise agreement between the rtPCR and culture as well as between the rtPCR and FAT detection methods. For both percent observed agreement and Cohen’s Kappa, the total samples included the combined 42 positive and 10 negative urine samples (52 total).

## 3. Results

**Figure 2:**
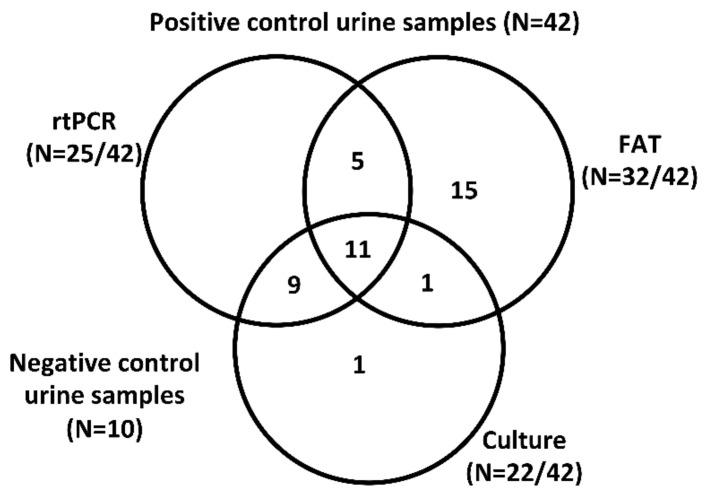
Venn diagram of test-positive bovine urine samples (N = 42 total). Numbers of bovine samples which were positive for *Leptospira* by FAT and/or culture and/or rtPCR are provided. Only 26% (11/42) samples were considered positive by all three assays.

Of the 42 bovine urine samples that were known to be positive for *Leptospira* by culture and/or FAT, 60% (25/42) were positive by rtPCR (Figure 2). Of 22 culture-positive samples, 91% (20/22) were rtPCR-positive and of 32 FAT-positive samples, 50% (16/32) were rtPCR-positive. Only 26% (11/42) of all samples were positive by culture, FAT and rtPCR. All negative controls were negative by rtPCR. The percent observed agreement for combined positive and negative results between rtPCR and culture results was 86.5% and 51.9% for rtPCR and FAT, respectively (Table 1). Cohen’s Kappa was performed to evaluate the levels of agreement between rtPCR and previously reported culture and FAT detection methods (Table 1). A Cohen’s Kappa value of 0.74 suggests substantial agreement between rtPCR and culture, while a Cohen’s Kappa value of 0.04 between rtPCR and FAT is indicative of only slight agreement, closely equivalent to random chance.

All rtPCR positive samples had a melting temperature (Tm) of 82.5 °C, equivalent to that which was previously determined for *L. borgpetersenii*, and in agreement with the previously determined species of *Leptospira* cultured from the urine of beef cattle [12,16]. All four rtPCR replicates for each sample were positive in 22/25 samples. In the remaining three samples, all of which were culture positive, two out of four replicates were positive in two samples and one sample had one out of four positive replicates. The rtPCR showed a relative specificity of 100% when compared with MAT-, FAT- and culture-negative samples. One sample that was culture-positive remained negative by FAT and rtPCR.

## 4. Discussion

Collectively, our results indicate that no single assay to detect leptospires in bovine urine samples is optimal. In this study, we consider sample collection and processing to be as close to ideal as possible in a non-experimental setting: urine was collected directly from the bladder by needle and syringe at the time of slaughter and immediately used for culture. Additionally, urine samples were transported to the laboratory at ambient temperature and processed for FAT and rtPCR within 3 h of collection. There are likely many reasons for the variability in positive results observed for each assay, including variability in composition of urine from outbred animals, as well as numbers of leptospires shed per mL of urine, as would be expected with field samples. Nevertheless, a side-by-side comparison of each technique with identical field samples highlights the presence of inherent variables and factors that can influence a test-positive result for the detection of leptospires in bovine urine samples; in several samples, the rtPCR was positive while FAT was negative, and conversely, when the rtPCR was negative, the FAT was positive. Such results emphasize the difficulty in defining a true reference test for field samples.

In our previous study [12], a primary goal was to obtain and identify bovine isolates of *Leptospira*, all of which were classified as *L. borgpetersenii*. Species identification is possible with cultured isolates but not by FAT. In the current study, the use of rtPCR provides a Tm value which was identical in all rtPCR positive samples, and to that previously established for *L. borgpetersenii*. Though culture is considered to be the gold standard diagnostic assay for the direct detection of *Leptospira*, it is inherently difficult, and performed only by specialist laboratories. Thus culture is limited in its application to field studies. The use of more than one assay to diagnose urinary shedding of leptospires in cattle is recommended.

## 5. Conclusions

The use of more than one diagnostic assay is recommended to detect and diagnose *L. borgpetersenii* in bovine urine.

## Figures and Tables

**Table 1:** Percent observed agreement and Cohen’s Kappa evaluation of the rtPCR method of detection compared to previously reported culture and FAT detection techniques. Reported values were calculated including both positive and negative testing (total of 52 samples).

		Culture	FAT
*rtPCR*	Percent Observed Agreement	86.5%	51.9%
		(20 + 25)/52	(16 + 11)/52
	Cohen’s Kappa Value	0.73	0.04
		Substantial Agreement	Slight Agreement
